# Supplementary material for: Karyological Diversification in the Genus Lyciasalamandra (Urodela: Salamandridae)
Source: Animals (Basel). 2021 Jun 8;11(6):1709. doi: 10.3390/ani11061709 (PMC8228943; doi:10.3390/ani11061709)
Supplement: Supplementary file 1 [file animals-11-01709-s001.zip › animals-1248105-supplementary.pdf]

Table S1. Chromosome morphometric parameters of the studied taxa. R.L. = Relative Lenght; C.I. = Centromeric Index; Sp. = Species; Chr. = Chromosome; Solid line boxes = homomorphic NORs; dashed line boxes = heteromorphic NORs

Sp./ Chr.	*L. a. antalyana*		*L.b. billae*		*L.f.flavimembris*		*L. helverveni*		*L. l. luschani*		*L. l. basoglui*		*L. l. finikensis*		*S. s. salamandra*		*S. s. gigliolii*		*S. lanzai*	
	R.L.	C.I.	R.L.	C.I.	R.L.	C.I.	R.L.	C.I.	R.L.	C.I.	R.L.	C.I.	R.L.	C.I.	R.L.	C.I.	R.L.	C.I.	R.L.	C.I.
1	12.4	48.4	12.6	46.3	12.5	47.9	13.3	48.0	13.0	46.5	12.8	44.9	12.5	48.4	12.0	49.0	12.3	43.8	13.9	48.2
2	11.4	43.9	11.4	41.5	11.2	42,8	11.6	42.7	11.8	42.5	11.9	46.5	11.5	43.9	11.3	43.9	11.6	43.5	12.9	47.3
3	11.0	43.7	11.2	42.6	11.2	46,4	11.0	41.5	11.3	41.6	11.2	41.7	11.0	43.7	11.1	43.7	11.1	48.3	11.6	46.3
4	10.7	48.7	10.7	44.4	10.8	45.3	10.9	43.2	10.0	44.7	10.7	44.7	10.5	48.7	10.9	48.7	10.9	46.7	10.1	46.2
5	9.8	46.9	10.0	42.8	9.6	42.7	9,4	44.8	9.7	42.9	9.1	46.8	10.0	46.9	10.1	46.9	10.9	49.3	9.2	39.6
6	9.5	31.1	9.4	32.4	9.4	30.8	9.2	29.2	9.2	32.4	9.0	31.5	9.2	33.0	9.4	31.9	9.7	40.6	8.1	28.0
7	8.3	41.5	8.6	40.3	9.0	41.2	8.2	41.9	8.3	40.1	8.2	40.5	8.2	28.5	8.2	41.5	7.2	41,7	8.0	46.9
8	7.4	28.9	7.4	28.5	7.6	29.0	7.5	30.4	7.5	33.5	7.6	29.4	7.7	33.4	7.6	40.4	6.3	31.8	7.1	32.0
9	5.8	44.0	5.4	46,7	5.4	43.0	5.2	46.1	5.5	42.9	5.4	44.0	5.5	44.0	5.6	44.0	5.8	46.4	5.4	40.8
10	5.3	39.3	5.3	41.3	5.1	42.4	5.2	42.4	5.3	40,4	5.2	39.3	5.3	39.3	5.4	39.3	5.2	40.0	4.9	43.8
11	4.4	40.2	4.2	40.0	4.3	39.7	4.5	41.3	4.5	40.6	4.9	42.3	4.6	42.3	4.6	40.2	4.9	42.8	4.7	40.9
12	4.0	34.1	3.8	30.1	4.0	35.1	4.0	32.1	3.9	34.1	4.1	33.1	4.0	30.1	3.8	33.9	4.1	35.2	4.3	31.4
